# Transient regulation of three clustered tomato class-I small heat-shock chaperone genes by ethylene is mediated by SlMADS-RIN transcription factor

**DOI:** 10.1038/s41598-017-06622-0

**Published:** 2017-07-25

**Authors:** Vijaya Shukla, Rakesh K. Upadhyay, Mark L. Tucker, James J. Giovannoni, Sairam V. Rudrabhatla, Autar K. Mattoo

**Affiliations:** 1 Sustainable Agricultural Systems Laboratory, USDA-ARS, Henry A. Wallace Beltsville Agricultural Research Center, Beltsville, MD 20705-2350 USA; 2 USDA-ARS Robert W. Holley Center and Boyce Thompson Institute for Plant Research, Cornell University campus, Ithaca, NY 14853 USA; 3 Department of Biology, Penn State University at Harrisburg, Middletown, PA 170-57 USA; 4 Soybean Genomics and Improvement Laboratory, Agricultural Research Service, United States Department of Agriculture, Beltsville, MD 20705 USA

## Abstract

Clustered class-I small heat-shock protein (sHSP) chaperone genes, SlHSP17.6, SlHSP20.0 and SlHSP20.1, in tomato are demonstrated to be transcriptionally regulated by ethylene during mature green (MG) fruit transition into ripening. These genes are constitutively expressed at MG fruit stage in two different tomato genotypes as well as in their ripening mutants, including rin, nor and Nr, and an ethylene-deficient transgenic line, ACS2-antisense. Notably, ethylene treatment of the MG fruit led to significant *sHSP* gene suppression in both wild-types, ACS2-antisense, *nor*/*nor* and *Nr*/*Nr*, but not the *rin*/*rin* mutant. Inability of ethylene to suppress *sHSP* genes in *rin*/*rin* mutant, which harbors *MADS-RIN* gene mutation, suggests that MADS-RIN transcription factor regulates the expression of these genes. Treatment of the wild type and ACS2-antisense fruit with the ethylene-signaling inhibitor, 1-methylcyclopropane (1-MCP), reversed the s*HSP* gene suppression. Transcripts of representative ethylene-responsive and ripening-modulated genes confirmed and validated sHSP transcript profile patterns. In silico analysis in conjunction with chromatin immunoprecipitation demonstrated MADS-RIN protein binding to specific CArG motifs present in the promoters of these chaperone genes. The results establish MADS-RIN protein as a transcriptional regulator of these chaperone genes in an ethylene-dependent manner, and that MADS-RIN protein-regulation of sHSPs is integral to tomato fruit ripening.

## Introduction

Small heat shock proteins (sHSPs) are ubiquitous ancient proteins with conserved structural features, which evolved before the divergence of Archaea, Bacteria, and Eukarya^1–7^. Research has established that sHSPs function as molecular chaperones, assisting protein folding and preventing aggregation of their target proteins^7–10^. Although sHSPs were initially discovered among up-regulated proteins during heat stress, it is now known that they are also constitutively expressed^10^. Human sHSPs – HSP20, HSP22 and HSP27 - have biological roles varying from muscle contraction and metabolism (HSP20), regulation of apoptosis and carcinogenesis (HSP22) to oxidative stress protection and cytoskeleton regulation (HSP27)^11^. In plants, diverse sHSPs are found with distinct subfamilies, and their up-regulation by heat has presented an avenue to study their role in thermotolerance^7^. They are also induced by other stresses^12^.

Among eukaryotes, sHSPs are more abundant in land plants, ranging in size from 12–42 kD^13^. Our interest in exploring regulation of sHSPs in plant biology, particularly during ripening of fruits, emanated from previous studies that identified *VISCOSITY 1* (*VIS1*), a tomato sHSP gene, as a regulator of pectin depolymerization effecting juice viscosity of the fruit^14^ and tomato sHSP21 as a stabilizer of photosystem II against oxidative stress and color change during tomato fruit ripening^15^. Moreover, a unique intron-less cluster of three sHSP chaperone genes, *SlHSP17*.*6*, *SlHSP20*.*0* and *SlHSP20*.*1*, is resident on the short arm of chromosome 6 in tomato and is differentially expressed during tomato fruit ripening^16^. Interestingly, the 5′ end of each of these *sHSP* genes is enriched in sequence motifs responsive to hormones, i.e., ABA, ethylene, GA, and methyl jasmonate^16^. The presence of ethylene-responsive elements in these *sHSP* genes suggests that ethylene may regulate them during fruit ripening. Another sHSP protein, *sHSP21*, was shown to regulate pigment development in tomato fruit^15^. Ethylene directly or indirectly promotes transcription/translation of numerous ripening-related genes, including those associated with cell wall breakdown, carotenoid biosynthesis, aroma development, pigment accumulation, fruit softening, and flavor^17, 18^.

Tomato is an excellent system to dissect ethylene-mediated regulation of genes during fruit ripening^19–24^. More so, since several ripening tomato mutants, namely, *rin*, *nor*, *Nr*, *alc*, *cnr*, *frm* and *DFD*, are a good resource for characterization of fruit ripening^19^. Subsequent to the discovery that the *rin* mutation encodes a MADS-box transcription factor that regulates ripening^25^, the RIN protein was found to regulate a number of other genes involved in plant development^25–28^. *Never-ripe 2* (*Nr-2*) mutation is a semi-dominant mutation in a tomato ethylene receptor orthologous to Arabidopsis *AtETR1* ethylene receptor^29^, while the *non-ripening* (*Nor*) mutation is linked to a change in the expression of a NAC transcription factor, both affect ethylene and ripening-dependent gene expression^30^.

A plethora of genes are known to regulate fruit ripening. To the best of our knowledge, little is known about how sHSPs are transcriptionally regulated during tomato fruit ripening. To gain an insight into possible ethylene-mediated regulation of three clustered tomato *sHSP* genes, we studied their gene expression in wild-type Ailsa Craig variety and its isogenic ripening mutants – *rin*/*rin*, *nor*/*nor* and *Nr*/*Nr*, and in the wild-type Ohio8245 processing tomato line and its transgenic line harboring an anti-sense *ACC synthase 2* gene (2AS-AS). Here, we present results demonstrating that the class 1 *sHSP* gene cluster on Chr. 6 is negatively regulated by ethylene prior to fruit ripening. Further, we demonstrate that all the three tomato *sHSP genes* (*17*.*6*, *20*.*0 and 20*.*1*) harbor functional and interactive RIN-binding, CArG motifs in their promoters. The relevance of these findings to the control of ripening is discussed.

## Materials and Methods

### Plant Materials

Mutant tomato (*Solanum lycopersicum cv* Alisa Craig) lines of *ripening-inhibitor* (*rin*), *non-ripening* (*nor*) and *Never-ripe* (*Nr*) were repeatedly backcrossed into the cultivar Ailsa Craig to obtain near isogenic lines for these mutants. Mutant and wild type (WT) Ailsa Craig and a previously characterized *ACC-synthase 2* (*ACS2*) silenced transgenic line (2AS-AS) in cultivar Ohio8245 and its azygous WT control^31^ were grown in a temperature-controlled greenhouse under natural light conditions. Tomato fruits at 5 days before breaker (−)5BR – which is equivalent to mature green (MG) stage, breaker (BR) and red ripe (7 days after breaker [BR + 7]) were analyzed by RNA gel-blot analysis. Fruits from the Ohio8245 WT and its 2AS-AS line were harvested at (−)5BR (MG stage), BR and BR + 8 and then treated and analyzed as described below.

### Ethylene and 1-MCP treatments

Mature Green [MG, (−)5BR] tomato fruits were used for ethylene and 1-methylcyclopropene (1-MCP) treatment in three independent biological replicates. Briefly, MG tomatoes were harvested from each line, wiped with 70% ethanol and then rinsed with double distilled water. For ethylene treatment, fruits were placed in a known volume of air-tight jars and sealed with rubber septum glued to the lid. Ethylene was injected into the jars with a syringe needle to a final concentration of 25 ppm. A separate batch of MG fruit harvested at the same time was treated with 2 ppm 1-MCP to inhibit the ethylene response - SmartFresh (AgroFresh, Collegeville, PA, USA) is a powder that includes 1-MCP at 3.3%. The powder releases MCP as a gas when added to water. SmartFresh, 20 mg, was mixed vigorously with 380 mg of sucrose (1:20 w/w). The sucrose diluted SmartFresh (2.5 mg L^−1^) was put into a 1.5 mL tube that was hung inside the closed container and 0.5 mL of water injected into the 1.5 mL tube through the septum in the lid of the container. Corresponding control fruits (air) were enclosed in a similar container without ethylene or 1-MCP treatment. Fruits were collected at 0, 12 and 24 hours of treatment, peeled and the pericarp immediately frozen in liquid nitrogen. For ethylene treatment of leaves, 8–10 fully expanded leaves harvested from mature Ailsa Craig plants were treated with 25 ppm ethylene for 0, 24, 48, 72 and 96 hours in the dark at 25 °C. At the indicated times, leaf samples were removed, immediately frozen in liquid nitrogen and then stored at −70 °C.

### Total RNA extraction, RNA gel blotting, cDNA synthesis and quantitative RT-PCR

Frozen tomato fruit and leaf samples were ground with liquid nitrogen to a fine powder. Total RNA was extracted from 100 mg tissue using Plant RNeasy kit according to manufacturer’s instructions (Qiagen), and treated with RNase-Free DNase (QIAGEN). Northern blotting was done as described previously^25^. A total of 2 µg RNA was used for cDNA synthesis using the iScript Advanced cDNA synthesis kit (Bio-Rad). cDNA was diluted 10-fold for further use. Quantitative Real-time PCR was performed using Sso Advanced Universal SYBR Green Supermix (Bio-Rad) in a Bio-Rad cycler (CFX96 Bio-Rad Real Time PCR machine). PCR conditions applied were: 95 °C for 10 min; 95 °C for 15 sec; and 60 °C for 60 sec (40 cycles), followed by melt curve analysis^32^. For relative quantification, Ct values of gene expression were quantified according to ∆∆C_T_ method^33^. Ct/Cq quantification cycle was calculated by following the Bio-Rad CFX Manager 3.1 based on the MIQE (Minimum Information for publication of Quantitative real-time PCR Experiments) guidelines^34^. Relative fold changes were calculated as previously described^35^. Tomato actin (*SlACT2*) was used as a constitutively expressed gene to normalize the expression of the target genes^32^. Accession numbers and primer sequences of genes analyzed are listed in Supplementary Table S1. Data represent the average ± standard error from a minimum of three independent biological replicates for each gene transcript profiled.

### *In silico* analysis of class I *sHSP* (*17*.*6*, *20*.*0 and 20*.*1*) promoters for RIN protein binding (CArG motifs) sites

Promoters (≈3 kb of the 5′ upstream sequence from the start codon, ATG) of all the three class I tomato *sHSPs*, *17*.*6*, *20*.*0 and 20*.*1*, were identified based on information from the International Tomato Genome Sequencing Consortium (SGN; solgenomics.net) database (version ITAG 2.4). Two databases, Plant CARE relational database^36^ and PLACE (the plant-*cis*-acting regulatory DNA elements) database^37^ were used to identify putative regulatory motifs in the *sHSP* promoters, including the potential RIN binding, CArG-box motif sequences [{C(C/T) (A/T)6(A/G)G}, {C(A/T)8G} and {C(C/T)(A/T)G(A/T)4 (A/G)G}]^38, 39^. The consensus sequence for the CArG boxes was analyzed using the WebLogo 3 program (http://weblogo.threeplusone.com/)^40, 41^. The CArG motifs found in the s*HSP* gene promoters are listed in Supplementary Table S2.

### Chromatin immunoprecipitation (ChIP) and quantification of enrichment via ChIP-qPCR

For chromatin cross-linking, breaker (BR) tomato fruit pericarp tissue was diced (1–2 cm^2^) and placed in a 50 mL falcon tube filled with 20 ml of 1% formaldehyde (Sigma). Vacuum infiltration was done at 62 cm of Hg for 10–15 min until air bubbles appeared in the formaldehyde solution. Cross-linking was stopped by adding 0.125 M glycine and vacuum infiltration continued for an additional 5 min. Cross-linked tissue was then rinsed thrice with water, frozen in liquid nitrogen and stored at −80 °C. For each ChIP reaction, 1 g of the cross-linked tissue was analyzed and 3 µL of RIN antibody was used^42^. A plant ChIP Kit (Epigentek) was used for ChIP assays according to manufacturer’s instructions with a few modifications^43^. Briefly, chromatin was isolated from 1 g of crosslinked tissue and then sheared by a 30 sec pulse at 30 second intervals for 10 cycles in a Branson 2200 water bath sonication machine at default settings (50/60HZ Power). The antibody coating step was performed at 4 °C for 8–10 h with anti-RIN antibody^25^. Rabbit IgG was used as negative control. Chromatin binding was carried out at 4 °C on an orbital shaker at 80–100 rpm for 8 h. Primers spanning each CArG motif present in each *sHSP* promoter (*17*.*6*, *20*.*0 and 21*.*0*) are listed in Supplementary Table S3. Promoter primer specificity was checked with blast program [International Tomato Genome Sequencing Consortium (SGN; solgenomics.net) database, version ITAG 2.4] and their specificity tested using genomic DNA as template with PCR. PCR products were checked on agarose gels, and single amplicons representing the desired size were obtained. These primers were used to assay for the enrichment of CArG motifs with quantitative real-time PCR following the PCR conditions described above. Percent input method [100*2^Cq^ (adjusted input) - Cq (IP)] was followed to calculate enrichment of fragments^44^ where n = 3 (three BR fruits from three different plants were used for chromatin immunoprecipitation to calculate the average and standard deviation in ChIP- q-PCR reaction).

### Data analysis and statistics

Statistical analysis was carried out using two-tailed student’s t-test with P values < 0.05 treated as statistically significant.

## Results

### Upregulation of Class-I *SlHSP17*.*6*, *SlHSP20*.*0 and SlHSP20*.*1* gene transcripts - transcriptomic and qRT-PCR analyses

**Figure 1:**
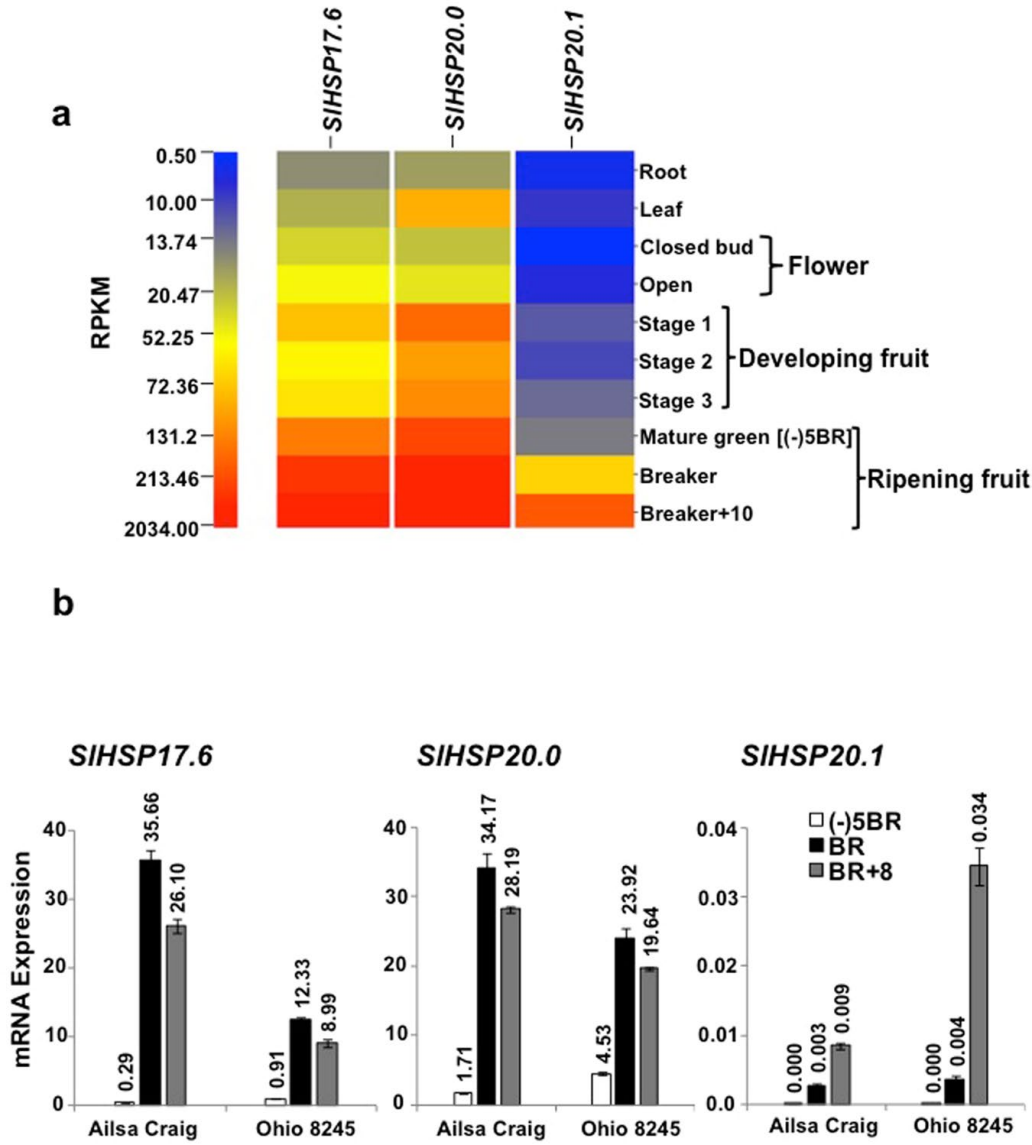
Transcriptome analysis and Q-PCR of class-I *SlHSP* genes (*17*.*6*, *20*.*0*, *20*.*1*) in wild type tomato during ripening. (**a**) RPKM values for the indicated *SlHSP* gene transcripts in tomato during plant growth and development and fruit ripening [root, leaf, bud, flower, 3 fruit developmental stages, mature green, breaker and red ripe (breaker + 10)] were derived from RNA-seq data in the SGN database (*Solanum lycopersicum* cv. Heinz). (**b**) Q-PCR analysis of *SlHSP* (*17*.*6*,*20*.*0 and 20*.*1*) genes was performed using total RNA isolated from *Solanum lycopersicum* cv. Ohio8245 and Ailsa Craig wild type fruits at the indicated ripening stages. A 10-fold diluted cDNA was used for transcript quantification using gene specific primers.

To gain insight into the expression of the three clustered *sHSP*’ genes (17.6, 20.0, 20.1) during tomato fruit ripening, available transcriptome data (*Solanum lycopersicum* cv. Heinz) was retrieved from International Tomato Genome Sequencing Consortium (SGN; solgenomics.net) database (version ITAG 2.4). RPKM (reads per kilobase of transcript per million mapped reads) data obtained for the three transcripts during plant development and fruit ripening are presented as heat map in Fig. 1a. Transcripts of all the three *SlHSP* genes are highly up-regulated during ripening, abundance of each increasing from MG to BR stage [7.28-fold for *SlHSP17*.*6*, 5.35-fold for *SlHSP20*.*0* and 17.40-fold for *SlHSP20*.*1*], with *SlHSP20*.*1* being more abundant at the BR + 8 stage (Fig. 1a). Based on these transcriptomic data, it is noted that these *sHSP* genes are less abundant in other plant parts and during tomato development relative to that in the fruit and during ripening. Quantitative real time PCR (Q-PCR) analysis of fruit RNA from two tomato varieties, namely, *S*. *lycopersicum* cv. Ohio8245 and Alisa Craig during ripening stages was performed for the three *SlHSP* genes for further validation. Results in Fig. 1b confirm ripening-regulated expression of the three *SlHSP* (*17*.*6*, *20*.*0*, *20*.*1*) genes in the processing tomato Ohio8245 variety as well as in Alisa Craig.

### The Class-I *SlHSP17*.*6*, *SlHSP20*.*0 and SlHSP20*.*1* gene transcripts were all more abundant in wild-type Ailsa Craig fruit than in the ripening mutants

**Figure 2:**
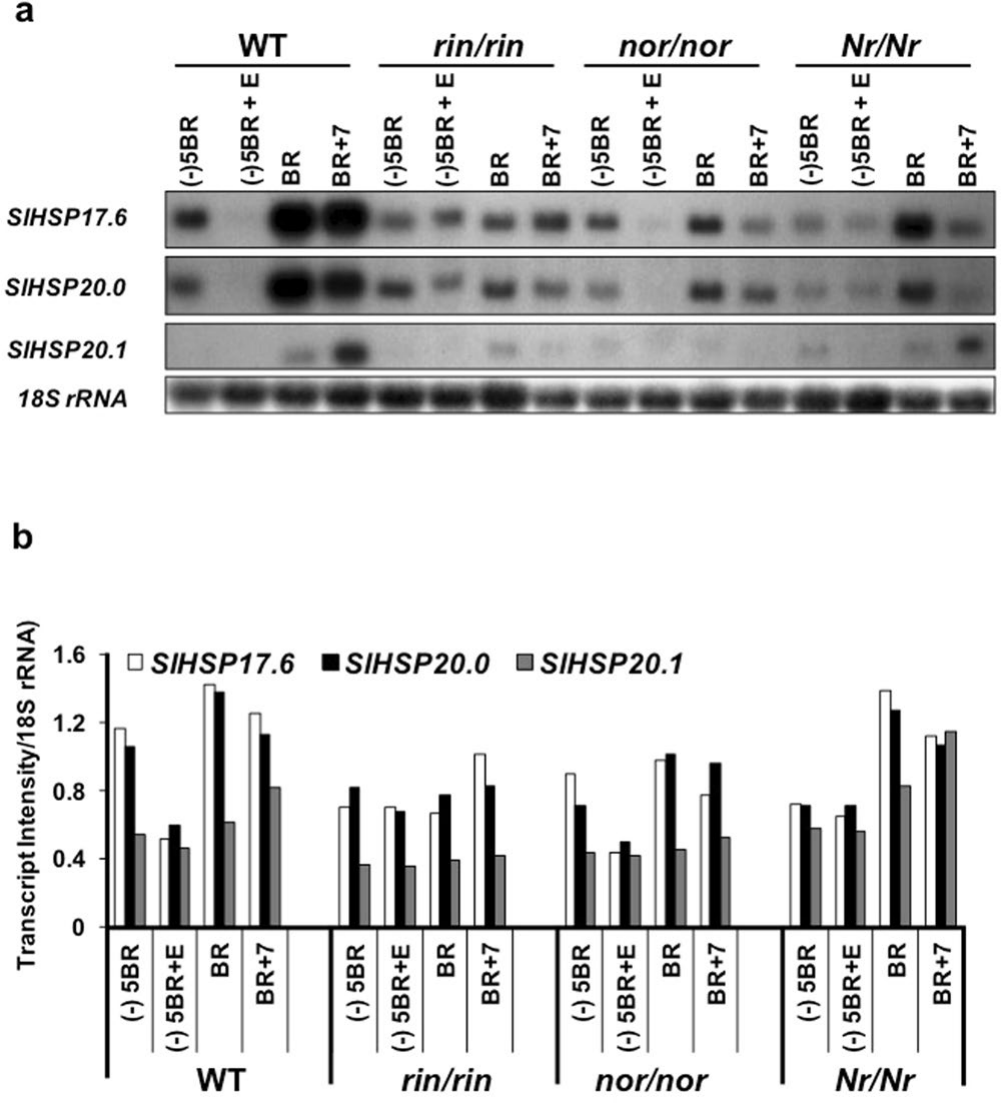
RNA blot-analysis of class-I *SlHSP* (*17*.*6*, *20*.*0* and *20*.*1*) genes in wild type (*Solanum lycopersicum* cv. Ailsa Craig) and its ripening mutants. (**a**) Fruits from wild type tomato var. Ailsa Craig along with near isogenic lines of *ripening-inhibitor* (*rin*/*rin*), *non-ripening* (*nor*/*nor*) and *Never-ripe* (*Nr*/*Nr*) mutant fruits were harvested at mature green [5 days before breaker (−)5BR)], breaker (BR) and red ripe (7 days after breaker, BR + 7) stages as described^25^. RNA was isolated, separated on gels, blotted and northern blot analysis carried out^25^, using gene specific probes as previously described^46^. Fruits at mature green stage (−)5BR from the indicated lines were given ethylene treatment and designated as (−)5BR + E. (**b**) Quantification of northern blot analysis using Image J program (https://imagej.nih.gov/ij/). The band intensity was calibrated with the band intensity of 18 S rRNA internal control used in northern blotting.

RNA gel-blot analysis of *HSP17*.*6*, *HSP20*.*0 and HSP20*.*1* genes in Ailsa Craig wild type **(**WT) and its near isogenic mutant lines, *ripening-inhibitor* (*rin*/*rin*), *non-ripening* (*nor*/*nor*) and *Never-ripe* (*Nr*/*Nr*), indicated differential expression and abundance during ripening (Fig. 2a,b). Abundance of *SlHSP17*.*6* and *SlHSP20*.*0* transcripts in the WT increased at the BR stage as compared to the MG fruit [(−)5BR], and remained more or less the same after 7 days (BR + 7; red ripe fruit) (Fig. 2a,b; WT, *SlHSP17*.*6*, *SlHSP20*.*0*). In contrast, *SlHSP20*.*1* transcripts were undetectable at (−)5BR stage, increased at BR and showed reasonable abundance at BR + 7 stage, but lower than that found for *SlHSP17*.*6* and *SlHSP20*.*0* transcripts (Fig. 2a,b; WT, compare *SlHSP20*.*1* with *SlHSP17*.*6* and *SlHSP20*).

In the *rin*/*rin* mutant, the abundance of *SlHSP17*.*6* and *SlHSP20*.*0* transcripts was apparent at (−)5BR but lower than the WT control, and remained more or less the same at BR and BR + 7 stages (Fig. 2a,b: *rin*/*rin*, *SlHSP17*.*6* and *SlHSP20*.*0*). In contrast, *SlHSP20*.*1* transcript abundance was drastically lower, with a minor but significant detection at BR stage (*rin*/*rin*, *SlHSP20*.*1*). In both *nor*/*nor* and *Nr*/*Nr* mutant fruit, abundance of *SlHSP17*.*6* and *SlHSP20*.*0* transcripts at the (−)5BR stage was lower than the WT control line, peaked at the BR stage and decreased thereafter; the abundance of *SlHSP20*.*1* transcripts in these two mutants was barely detectable and no different than that in the *rin*/*rin* mutant (Fig. 2a,b; *nor*/*nor*, *Nr*/*Nr*). Since these mutants are deficient in ethylene production, it was surmised that the candidate class-I *SlHSP* gene transcript expression is regulated by ethylene.

### Exogenous ethylene application suppresses *SlHSP* gene transcripts in MG fruit of WT, *nor*/*nor* and *Nr*/*Nr* but not in *rin*/*rin* mutant line

To delineate which genetic locus (considering the three mutants) regulates the expression of class-I *sHSP* genes, the (−)5BR (MG fruit) of WT and mutant lines were held in air-tight desiccators with 25 ppm ethylene (abbreviated as E) for 15 h, and the abundance of *SlHSP17*.*6*, *SlHSP20*.*0 and SlHSP20*.*1* transcripts was analyzed by RNA gel-blot analysis. Surprisingly, transcripts of all the three *SlHSP* genes in Ailsa Craig WT as well as in *nor*/*nor* mutant fruit were found suppressed by ethylene treatment (Fig. 2a,b; WT and *nor*/*nor*, compare lane [−5BR + E with lane (−)5BR], and slightly in the *Nr*/*Nr* mutant [*Nr*/*Nr*, compare lane (−)5BR + E with lane (−)5BR]. However, ethylene treatment did not elicit inhibition of *SlHSP17*.*6* and *SlHSP20* transcripts in the *rin* mutant [Fig. 2; *rin*/*rin*, compare lane (−)5BR + E with lane (−)5BR]. These results indicated that RIN is likely associated with ethylene-induced suppression of class-I *SlHSP17*.*6* and *SlHSP20*.*0* chaperone gene transcripts.

### Quantitative-Real Time PCR analysis confirms ethylene-mediated suppression of class-I *SlHSP* genes in wild type tomato and its anti-ACS2 homozygous transgenic line

**Figure 3:**
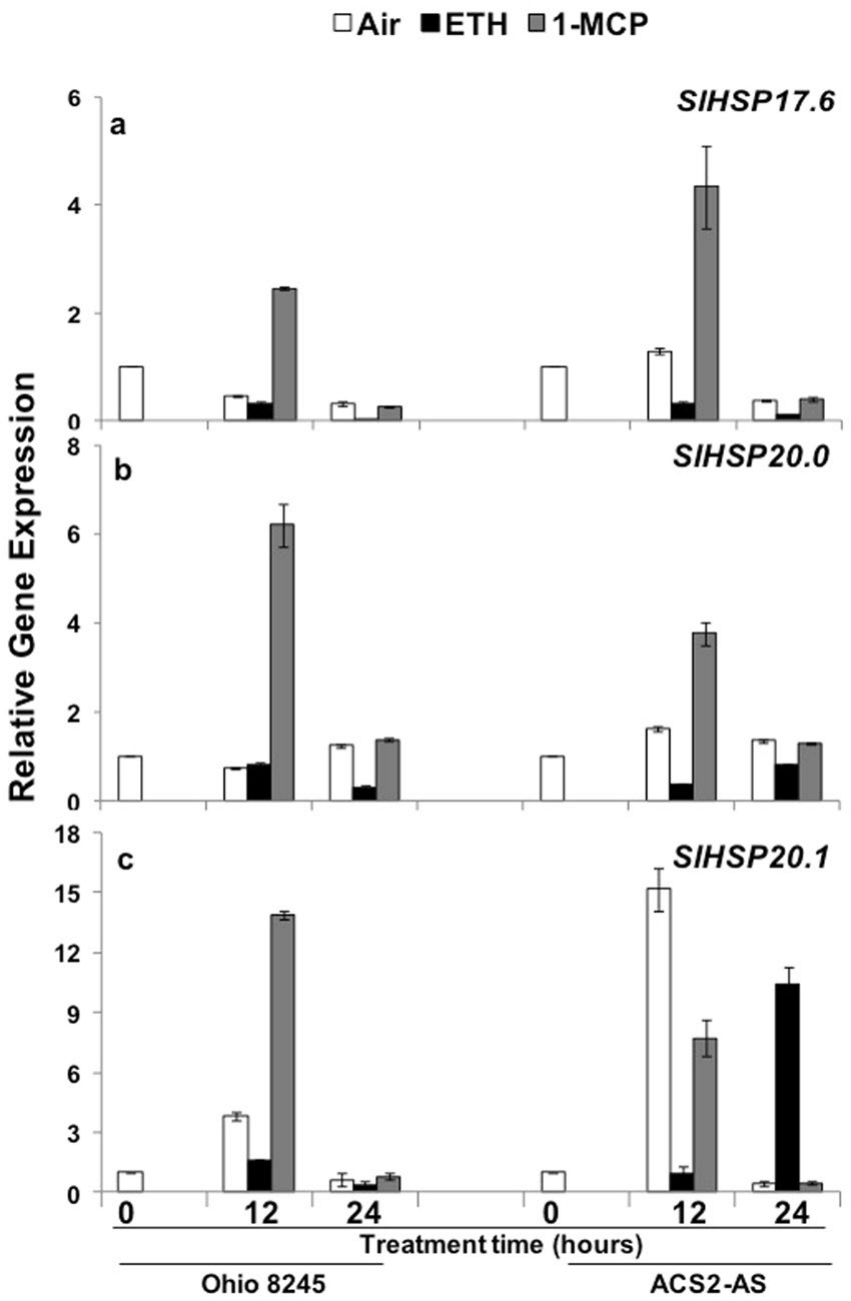
Quantitative RT-PCR expression analysis of class-I *SlHSP* (*17*.*6*, *20*.*0 and 20*.*1*) genes in wild type tomato (*Solanum lycopersicum* cv. Ohio8245) and ethylene-deficient genotype (ACS2-AS). Fruits at mature green stage from two genotypes (WT - Ohio8245) and ethylene-deficient genotype (ACS2-AS) were treated with ethylene(ETH), 1-MCP (1-MCP), or left in air. Fruit RNA was isolated from three independent biological samples and Q-PCR analysis was carried out as described in the Materials and Methods section. Error bars indicate standard deviation of data from a minimum of three replicates.

For further studies on ethylene regulation of class I *SlHSP17*.*6*, *SlHSP20*.*0 and SlHSP20*.*1* gene transcripts, we employed the Ohio8245 processing variety of tomato and its antisense-ACS2 transgenic line (designated as ACS2-AS, which is constitutively deficient in the *in vivo* ethylene production)^31^. MG fruit from Ohio8245 and the ACS2-AS homozygous line were incubated separately with ethylene or 1-MCP (inhibitor of ethylene signaling) or left in the air as described in the Materials and Methods section. At time 0, 12 and 24 h after treatment, fruit were sampled, their pericarp RNA isolated, and abundance of the *SlHSP* gene transcripts analyzed by Q-PCR. Expression of *SlHSP17*.*6* was lower at 12 h of treatment with ethylene but higher in 1-MCP-treated Ohio8245 compared to samples in air, and by 24 h *SlHSP17*.*6* expression was further suppressed in ethylene-treated samples (Fig. 3a, *SlHSP17*.*6*). This trend in *SlHSP17*.*6* transcript levels was mimicked in the ACS2-AS, ethylene-deficient fruit, which registered much higher suppression in the ethylene-treated fruit (Fig. 3a, ACS2-AS). Interestingly, *SlHSP17*.*6* and *SlHSP20*.*0* transcript abundance was found decreased also in ethylene-treated tomato leaves for up to 96 h (Supplementary Fig. S1).

In the case of *SlHSP20*.*0* expression, suppression by ethylene was apparent at 24 h of ethylene treatment in both Ohio azygous line and ACS2-AS fruits, while 1-MCP-treated fruits had higher expression. Moreover, ethylene inhibition of these transcripts was clearly seen early at 12 h of treatment (Fig. 3b, *SlHSP20*.*0*). A similar trend of ethylene suppression was found for *SlHSP20*.*1* at 12 h with elevated expression in the 1-MCP-treated fruit (Fig. 3c, *SlHSP20*.*1*). However, ethylene-mediated suppression of *SlHSP20*.*1* transcripts in ACS2-AS line was reversed in 24 h-treated fruit, with 1-MCP treatment blocking this up-regulation. It appears that the suppression of *SlHSP20*.*1* transcripts in ethylene-deficient ACS2-AS fruit on prolonged incubation (i.e., 24 h) may require a higher ethylene dose. However, this observation needs to be followed further in future experiments. These results confirm that ethylene suppresses these three class-I *SlHSP* genes.

### Validation of ethylene and 1-MCP treatment effects by testing expression analysis of known ethylene-responsive genes

**Figure 4:**
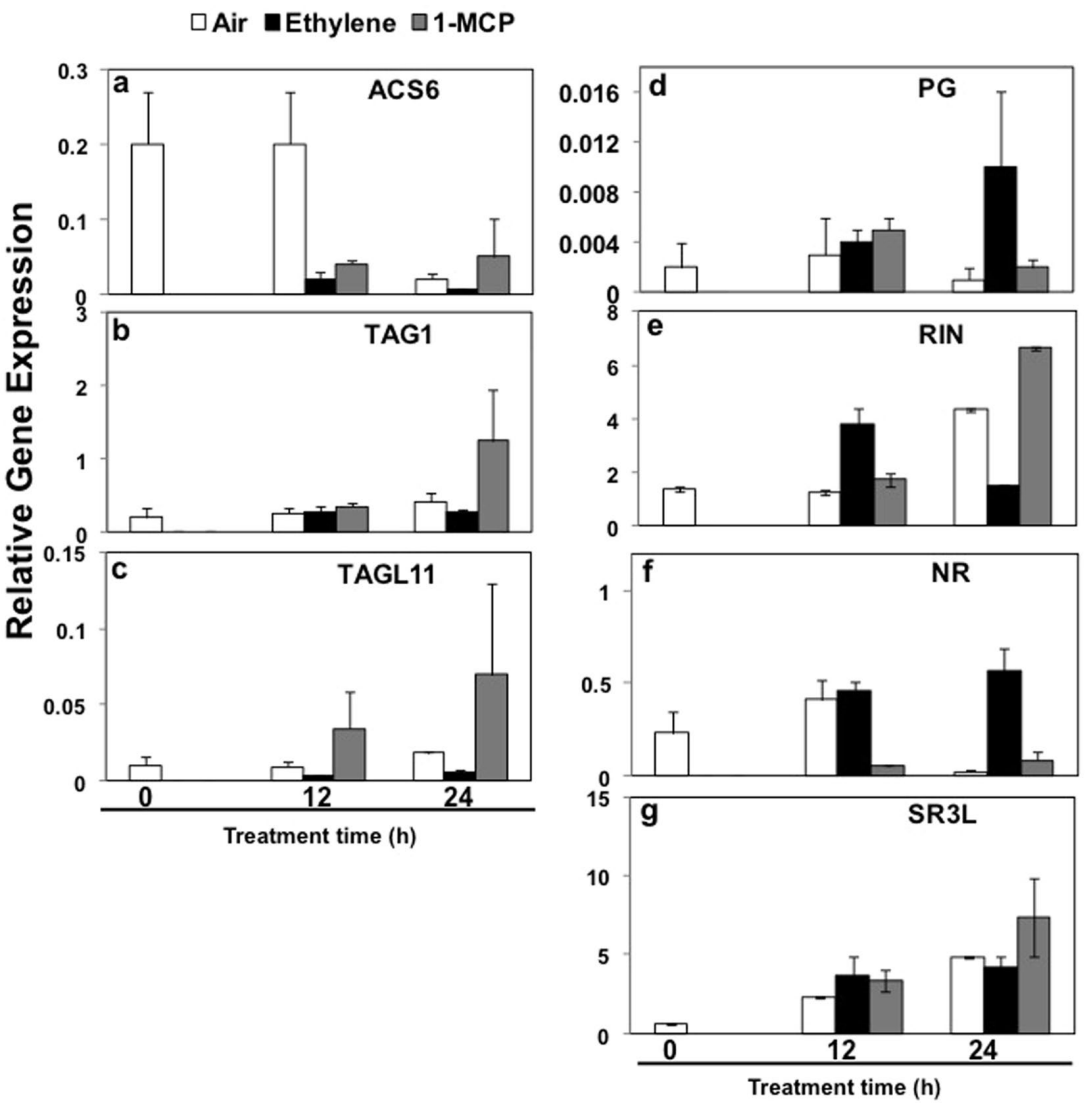
Quantitative RT-PCR analysis of ethylene responsive and nonresponsive genes in ethylene-treated wild type Ohio8245 tomato fruits. Fruits from wild type (*Solanum lycopersicum* cv. Ohio8245) control were exposed to air, ethylene or 1-MCP as described in the Materials and Methods section. RNA was isolated and transcripts for *ACS6*, *TAG1*, *TAG11*, *PG*, *RIN*, *NR* and *SR3L* genes were quantified by Q-PCR. Error bars indicate standard deviation from a minimum of three replicates.

To insure reliable ethylene and 1-MCP treatment and thus accurate interpretation of our *SlHSP* gene expression results, we evaluated the expression of 7 additional genes previously shown to be regulated by ethylene (Fig. 3). *ACS6* suppression by ethylene and up-regulation by 1-MCP treatment was previously shown^45^. Similar to these effects, *ACS6* expression was found highly suppressed in ethylene-treated Ohio azygous fruit but was elevated in 1-MCP-treated fruit, particularly in the 24-h samples (Fig. 4a). *TAG1* is also known to be slightly suppressed in response to exogenous ethylene^46^. This too was found to be the case in our experiments, with 1-MCP treatment giving an elevated *TAG1* expression in 24-h samples (Fig. 4b). Data on *TAGL11* expression indicated clear effects both with 12-h and 24-h treatments. Specifically, in the ethylene-treated fruit, *TAGL11* was suppressed while in 1-MCP-treated fruit its levels were elevated, indicating that ethylene suppresses *TAGL11* transcript (Fig. 4c). *PG1*, *Polygalacturonase 1*, is known to be induced by exogenous ethylene^47, 48^. This was evident in 24-h ethylene-treated samples and reflected by being inhibited in MCP-treated fruit (Fig. 4d). *RIN* was shown to be slightly up-regulated by exogenous ethylene^25^. Also, as expected, *RIN* transcripts were abundant within 12-h exposure of control fruit to exogenous ethylene and 1-MCP inhibited their expression (Fig. 4e). However, by 24 h of treatment, while air-exposed fruits had elevated *RIN* transcripts, ethylene-stimulation was already found reduced, which was in concurrence with stimulation in the presence of 1-MCP (Fig. 4e). *NR* gene transcripts are also induced by exogenous ethylene^49^, and likewise, *NR* transcripts accumulated in response to ethylene while 1-MCP suppressed its expression, indicating *NR* as an ethylene-inducible gene (Fig. 4f). *SR3L* is a calcium-signaling gene regulated by RIN and ethylene during ripening^50^. In our experiment, this gene was slightly up-regulated by ethylene treatment at 12 h but the up-regulation was not significantly different than in air at 12 h or 24 h (Fig. 4g). These data confirmed that our hormone and inhibitor treatment protocols delivered gene transcription data in line with those reported previously.

### *In silico* analysis of promoters of *SlHSP17*.*6*, *SlHSP20*.*0* and *SlHSP20*.*1* genes reveal putative RIN binding sites

**Figure 5:**
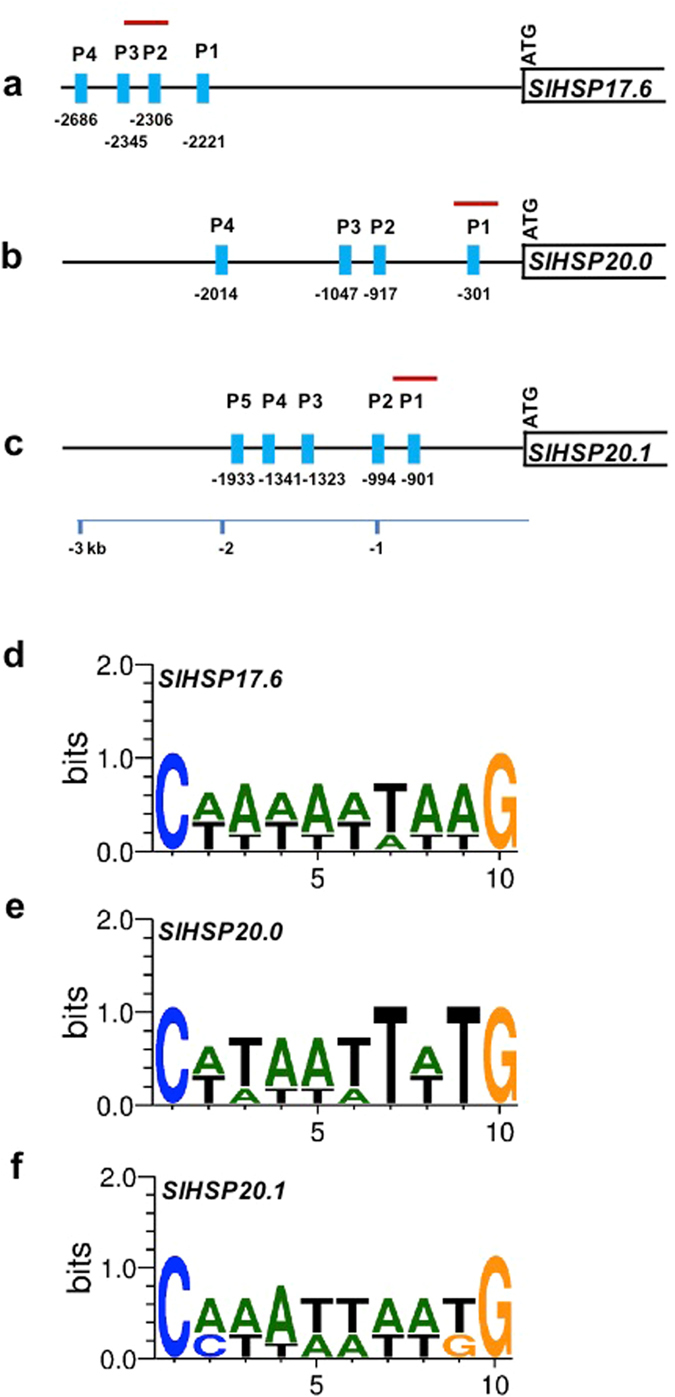
*In silico* analysis of class-I *SlHSP* (*17*.*6*, *20*.*0*, *20*.*1*) gene promoters. Promoter regions (≈3 kb of the 5′ upstream region of the start codon) of tomato *SlHSP17*.*6*, *SlHSP20*.*0 and SlHSP20*.*1* genes were extracted using the International Tomato Genome Sequencing Consortium (SGN) database (version ITAG 2.4). Two databases, Plant CARE relational database^35^ and PLACE, the plant-*cis*-acting regulatory DNA elements database^25^ were used for plant *cis-*element search in the promoters of the described *SlHSP* genes. The possible RIN binding CArG-box motif sequences are [{C(C/T) (A/T)6(A/G)G}, {C(A/T)8G} and {C(C/T)(A/T)G(A/T)4 (A/G)G}]^38, 39^. All the CArG motifs found in *SlHSP* gene promoters are listed in Supplementary Table S2. The promoter position(s) significantly enriched in ChIP assay are highlighted with horizontal red line. (**a**) *SlHSP17*.*6* gene promoter contains CArG *cis* element positions (here denoted as ‘P’), respectively at P1 (−2221), P2 (−2306), P3 (−2345) and P4 (−2686). (**b**) *SlHSP20*.*0* gene promoter contains four CArG motifs positioned at P1 (−303), P2 (−917), P3 (−1047) and P4 (−2014), respectively. (**c**) *SlHSP20*.*1* gene promoter harbors five CArG *cis* elements, positioned at P1 (−901), P2 (−994), P3 (−1323), P4 (−1341) and P5 (−1933). (**d**,**e**,**f**) show corresponding conserved locations and their distribution patterns in the 10 bp consensus sequence described in the text.

Next, we analyzed the 3 Kb 5′ promoter regions of tomato *SlHSP17*.*6*, *SlHSP20*.*0* and *SlHSP20*.*1* genes for the presence of RIN binding ‘CArG’ motifs using PLACE^37^ and Plant Care databases^36^. All the three *SlHSP* genes were found decorated with the CArG RIN binding motif, with *SlHSP17*.*6* and *SlHSP20*.*0* each having 4 atypical motif types and *SlHSP20*.*1* having 3 ‘atypical’ and 2 possible motif types (Supplementary Table S2). CArG *cis* element positions (denoted as ‘P’) for *SlHSP17*.*6* were resident at −2221 (P1), −2306 (P2), −2345 (P3) and −2686 (P4) (Fig. 5a), for *SlHSP20*.*0* at −303 (P1), −917 (P2), −1047 (P3) and −2014 (P4) (Fig. 5b), and for *SlHSP20*.*1* at −901 (P1), −994(P2), −1323 (P3), −1341 (P4) and −1933 (P5) (Fig. 5c), respectively. Each of the four CArG *cis-*elements in the promoter of *SlHSP17*.*6* and *SlHSP20*.*0* genes fall in the category of ‘atypical’ [C(A/T)_8_G] *cis* elements, while in the promoter of *SlHSP 20*.*1*, three represent ‘atypical’ [C(A/T)_8_G] and the other two as ‘possible’ [C(C/T)(A/T)_6_(A/G)G] as previously noted^28, 38, 39, 51^.

For prediction of the *in vivo* RIN binding CArG-box motif, we analyzed the collected CArG-box sequences from all the three class-I *SlHSP* gene promoters which yielded a 10-bp motif consensus sequence, C(T/A/C)(A/T)_6_(A/T/G)G (Fig. 5d,e,f). No conservation was observed in the flanking regions upstream and downstream of the 10-bp core. The presence of CArG *cis* elements in *SlHSP* genes suggested the possibility that they could be targets of MADS-box *RIN* transcription factor binding as previously characterized for other genes^28, 42^.

### Chromatin immunoprecipitation (ChIP) identifies CArG motifs in the *SlHSP17*.*6*, *SlHSP20*.*0* and *SlHSP20*.*1* chaperone genes – confirmation that these are targets of SlMADS-RIN protein

**Figure 6:**
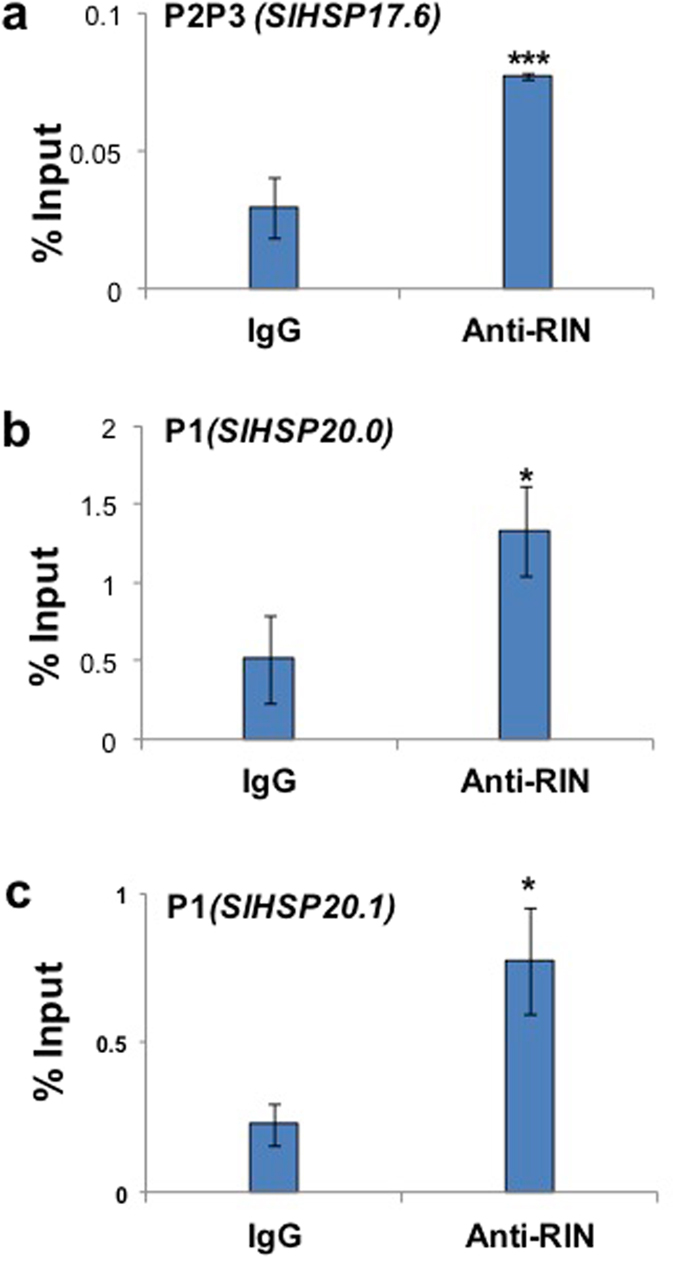
Chromatin immunoprecipitation (CHIP) and quantification of enrichment using CHIP-qPCR. Breaker stage Ailsa Craig tomato fruits were analyzed for chromatin cross linking. Plant CHIP Kit (Epigentek) was used for CHIP assays according to manufacturer’s instructions with few modifications^43^. Graph bars represent the relative DNA amounts of CArG-box sequences in the CHIP DNA recovered using either anti-RIN antibody or IgG antibody to those in the total input chromatin DNA. Data are the means of three independently prepared chromatin samples. Error bars indicate the standard deviation of each mean where n = 3. Rabbit IgG was used as a negative control. Asterisks indicate statistically significant differences, P < 0.05.

Each CArG motif present in the promoter of the three *SlHSP* genes (Supplementary Table S2 and Fig. 5) was tested for binding to the RIN protein using a chromatin immunoprecipitation (ChIP) assay and an anti-RIN antibody^42^. The forward and reverse primer pairs spanning these *cis* motifs were designed (Supplementary Table S3) and tested with a nucleotide blast search in Solanaceae Genome Network (SGN) database for their unique and single hits. None of the primer pairs gave non-specific blast hits in SGN database. Further, single amplification product was obtained with the synthesized primers when tested with genomic DNA for their specificity. All CArG motifs were assayed for probable ChIP enrichment. Two positions from the *SlHSP17*.*6* promote*r*, P2 (−2306) and P3 (−2345), were significantly enriched (P < 0.001) for the corresponding CArG *cis* element (Fig. 6a), whereas P1 position (−303) in *SlHSP20*.*0* promoter was also significantly enriched (P < 0.03) (Fig. 6b). P1 (−901) position in *SlHSP20*.*1* promoter was enriched with a significance P < 0.05 (Fig. 6c). The other predicted CArG *cis* element positions did not show significant enrichment in the ChIP assay. These data provide for the first time evidence that RIN protein interacts with the promoters of class-I *SlHSP* chaperone genes and mediates negative regulation of ethylene action.

## Discussion

We demonstrate here that ethylene and transcription of a subset of class-1 small heat-shock protein genes are interlinked, particularly at the initial phase of fruit ripening in tomato. Ethylene transiently suppressed *SlHSP* transcripts in two different tomato cultivars (Ailsa Craig and Ohio8245), A. Craig *nor*/*nor*, A. Craig *Nr*/*Nr* mutant and anti-ACS2 transgenic line in Ohio8245 background, but not in A. Craig *rin*/*rin* mutant. The insensitive nature of the *rin* ripening-deficient mutant to ethylene in modulating transcription of *HSP* genes suggests that they are associated with fruit ripening and regulated by ethylene albeit in a transient manner. The *SlMADS-RIN* transcription factor is known to regulate multiple processes involved in tomato fruit ripening^42^. Its mutation results in down regulation of many genes causing typical non-ripening phenotype beyond breaker stage^28, 51^. CArG *cis* elements are a common target of MADS box transcription factors (RIN protein) for regulating plant growth and development^38, 39, 52^. Interestingly, the promoter of each of the three *SlHSP* genes, *SlHSP17*.*6*, *SlHSP20*.*0* and *SlHSP20*.*1*, are decorated with multiple CArG *cis* elements, and we demonstrated RIN-specific binding to a subset of these CArG motifs. Thus, RIN protein seems likely involved in the regulation of these *SlHSP* genes. *SlHSP90* was previously reported to be a probable target of RIN protein^28^, and as shown here RIN protein has a larger regulatory network, which includes at least (but not limited to) three small heat shock protein genes. These class-I small heat shock proteins are encoded by nuclear genes and are resident in the chloroplast. It is noted here that ethylene-mediated and RIN-regulated transcriptional control for any chloroplast localized small heat shock protein has not been previously demonstrated. Additionally, this demonstrates a possible role of chloroplast localized proteins in tomato fruit ripening.

The class-1 s*HSP* genes studied here are basically intronless and uniquely clustered in a tandem repeat manner on the short arm of chromosome 6 in tomato^16^. Their differential expression during fruit ripening and the presence of many *cis* elements on their 5′ flanking region including motifs for ethylene perception are indicative of ethylene regulation^1, 6^. Previous studies with another class-I s*HSP* gene, *HSP21*, demonstrated its protein to function in plastid development (i.e., chloroplast to chromoplast transformation), protection of photosystem II under oxidative stress^15^ and heat stress^53^. In this regard, it is noted that *Arabidopsis* ethylene signaling mutants (*ein2* and *etr1*) were found defective in basal thermotolerance but accumulated *HSP101* and s*HSP*, suggesting that ethylene signaling may play a role in the thermal behavior of plants^54^. Although we have not pinned down the exact function of the intronless *SlHSP* genes, their expression patterns and ethylene regulation presented here implicate them in the ripening of tomato.

Small heat shock proteins have dynamic protein structure with diverse evolutionary origin and have been implicated not only in human disease but also stress acclimation in plants and other organisms^7, 55^. Therefore, ethylene-induced transient suppression of *HSP* genes mediated by RIN protein highlights their fundamental importance in plant biology, particularly fruit ripening. Our studies provide impetus to investigate yet unknown functional interactions of ethylene and *HSP* gene expression during fruit ripening. The transient nature of such a phenomenon could well indicate a role in the fruit’s transition from growth to ripening. Reports of transient suppression of a gene have appeared in the literature, each resulting in a specific function. For example, transiently regulated Rd22 gene expression in ABA-entrained plants indicated a probable memory in plants due to stress^56^. Similarly, cyanide produced concomitantly with ethylene biosynthesis results in transient transcriptional regulation of the cyanide-detoxifying gene, CYS-C1, in Arabidopsis, which then resulted in a cascade of downstream processes^57^. In addition, cell speciation in development is known to involve gene-regulatory responses to transient signals. Another example involves the transient accumulation of auxin which activates self-sustaining or hysteretic feedback system and results in unequivocal developmental responses^58^. Similarly, transient suppression of host gene expression due to viral infections in plants is a common phenomenon for viruses to establish the infection. PSbMV virus is known to transiently suppress the expression of host genes and inhibit host protein accumulation^59^.

**Figure 7:**
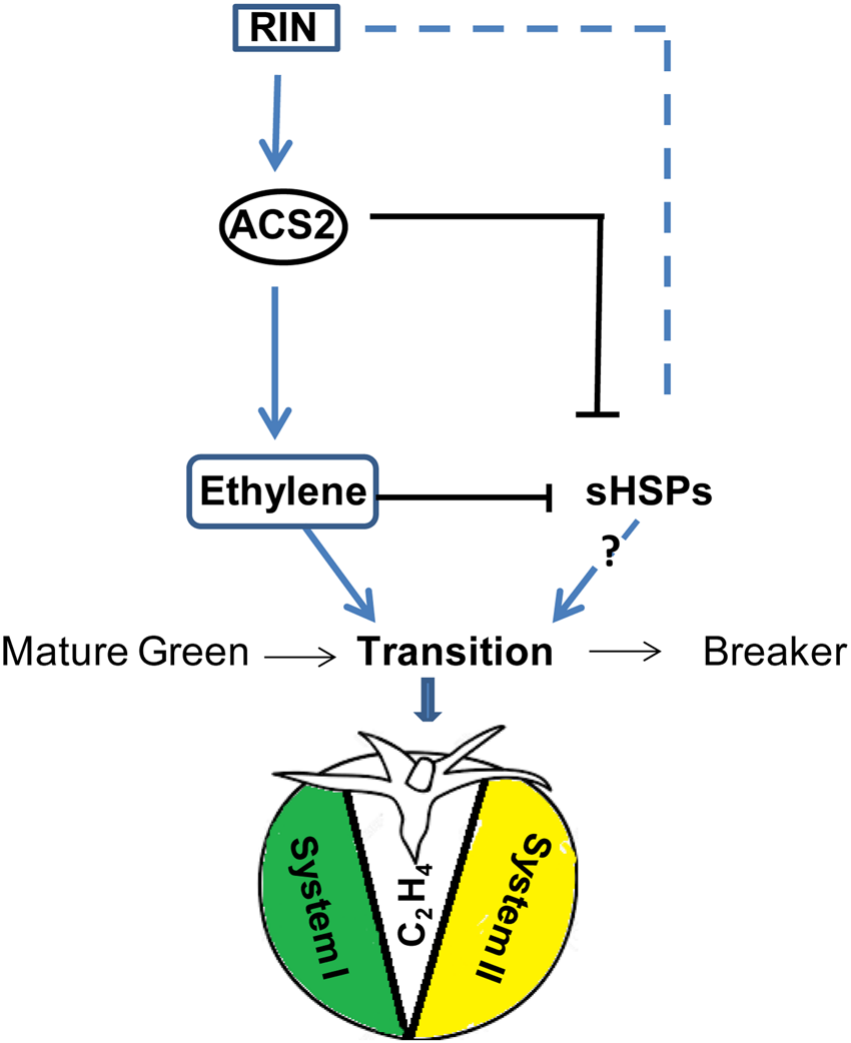
A schematic illustration showing additions to the previous fruit ripening model^60^ modified to include the *SlHSP* (*17*.*6*, *20*.*0*, *20*.*1*) genes with their proposed role in the transition from mature green to ripening phase of tomato fruit. RIN regulates ethylene biosynthesis in ripening tomato fruit involving ACS2 transcription (pointed blue arrows). During System1 ripening, low levels of auto-inhibitory ethylene are synthesized via *SlACS1A*,*6* and *SlACO1*,*3*,*4* ^60^. At the transition stage, the ripening regulator RIN plays a critical role, where *SlACS4* is induced to initiate a large increase in auto-catalytic ethylene that negatively feeds back on System I. *SlACS2*,*4* and *SlACO1*,*4* are involved in high ethylene production through System II^60^. *SlHSP* genes (sHPSs) are transiently suppressed by ethylene at the ripening-transition mediated by the RIN protein (blunt headed black arrows). Ethylene and *ACS2* exert negative regulation of the three class-1 *SlHSP* gene transcripts via interactions involving *RIN* protein (blue dashes).

Based on the above literature and data presented here, we hypothesize that ethylene-mediated transient down regulation of *SlHSP* genes at the onset of fruit ripening, when ethylene is just synthesized, is required for uninterrupted ripening. This contention is in tune with our demonstration that RIN regulates *SlHSP* gene transcription and the fact that *RIN* expression occurs at the onset of ripening of tomato fruit^35^. A model on the transcriptional regulation that interfaces ethylene, RIN and class I *SlHSPs*, including possible components involved, is presented in Fig. 7. This model builds on and shares some features with a previous fruit ripening model^60^. ACS2 regulates ethylene biosynthesis during fruit ripening and RIN is upstream of ethylene since RIN mutation abolishes ripening by inhibiting ethylene biosynthesis (pointed arrows) and ethylene inhibits *SlHSP* gene expression. A stronger inhibition of *SlHSP* gene expression in the ethylene-deficient ACS2-AS transgenic line adds support to the suggestion of an inhibitory role of ACS2 in regulating expression of the *SlHSP* genes (Fig. 7, blunt end arrows). Moreover, it is noted that the expression of *SlHSP* genes was not inhibited in the ethylene-treated RIN mutant, suggesting that RIN protein regulates the transcription of these genes. RIN mutant fruit does not ripen and thus RIN may primarily function at the transition stage at the onset of ripening. The data presented here implicate ethylene as a transcriptional regulator of *SlHSPs* genes in the fruit transition process from mature green to ripening. Further functional genomic studies are needed to characterize *in planta* the promoters of the *sHSP* genes studied here and use transgenic approaches to shed light on their specific role(s) in fruit physiology and ripening.

## Electronic supplementary material

Supplementary File
