# Supplementary material for: Transient regulation of three clustered tomato class-I small heat-shock chaperone genes by ethylene is mediated by SlMADS-RIN transcription factor
Source: Sci Rep. 2017 Jul 25;7:6474. doi: 10.1038/s41598-017-06622-0 (PMC5527083; doi:10.1038/s41598-017-06622-0)
Supplement: Supplementary file 1 — Supplementary File [file 41598_2017_6622_MOESM1_ESM.pdf]

Supplementary file

Transient regulation of three clustered tomato class-I small heat-shock chaperone genes by ethylene is mediated by SlMADS-RIN transcription factor

Vijaya Shukla[ac]*, Rakesh K. Upadhyay[ac]*, Mark L. Tucker[d], James J. Giovannoni[b], Sairam V. Rudrabhatla[c], Autar K. Mattoo[a1]

[a]Sustainable Agricultural Systems Laboratory, USDA-ARS, Henry A. Wallace Beltsville Agricultural Research Center, Beltsville, MD 20705-2350 (VS, RKU, AKM);

[b]USDA-ARS Robert W. Holley Center and Boyce Thompson Institute for Plant Research, Cornell University campus, Ithaca, NY 14853 (JJG);

[c]Department of Biology, Penn State University at Harrisburg, Middletown, PA 170-57 (RKU, VS) ;

[d]Soybean Genomics and Improvement Laboratory, Agricultural Research Service, United States Department of Agriculture, Beltsville, MD 20705 (MLT)

* Co-first authors

[1]Corresponding author: Autar K Mattoo. E-mail: autar.mattoo@ars.usda.gov

Supplementary Figure S1.

Quantitative RT-PCR expression analysis of class-I *SlHSP (17.6, 20.0 and 20.1)* genes in ethylene-treated leaves of wild type tomato (*Solanum lycoersicum* cv. **Ailsa Craig).** Fully expanded leaves were harvested from mature Ailsa Craig plants and treated with 25 ppm ethylene for 0, 24, 48, 72 and 96 hours. RNA isolation and cDNA preparation methodologies were the same as described in the Materials and Methods section. For Q-PCR, a 10-fold diluted cDNA was used for transcript quantification using gene specific primers.

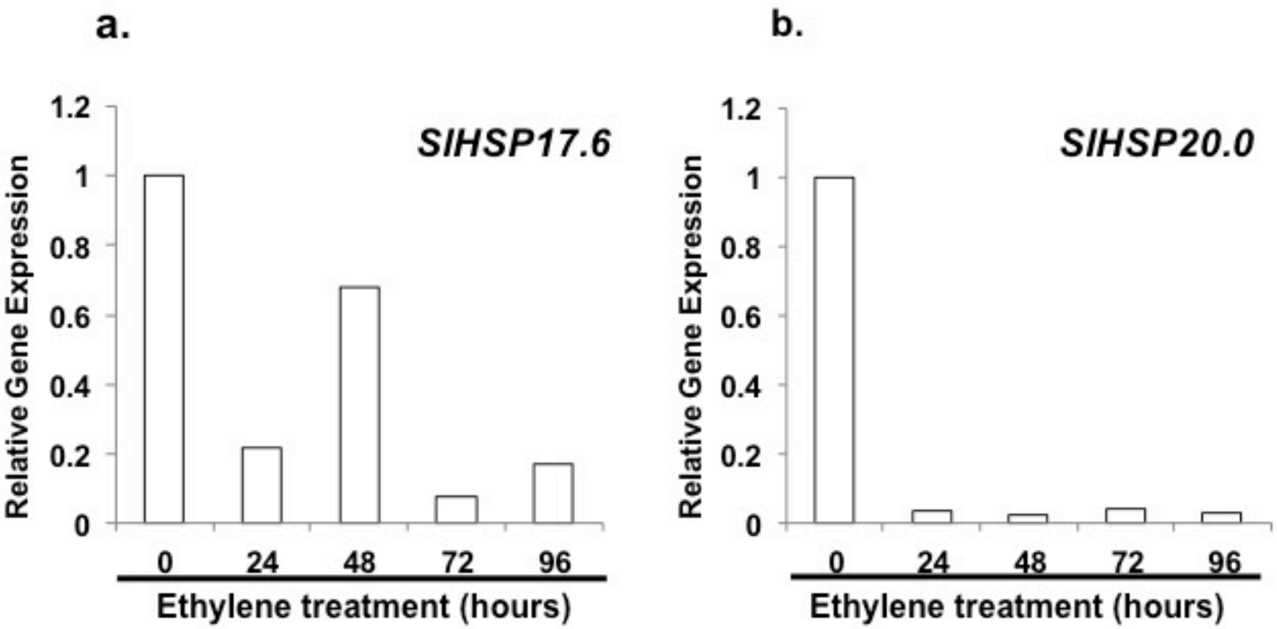

Supplementary Table S1.
List of genes and their primer sequences used for quantitative real–time PCR analyses

Abbreviated Name	Gene Bank ID	SGN Gene ID	Forward Primer (5′-3′)	Reverse Primer (5′-3′)
Actin	BT012695	Solyc04g011500.2.1	TGGTCGTACCACCGGTATTGTG	AATGGCATGTGGAAGGGCATAC
SlHSP17.6	AY150039	Solyc06g076560.1.1	AGGTCAAGTCCATTGAGATTTCTG	ACAGAGCAACTTCAAACCTTCAAC
SlHSP20.0	AJ225048	Solyc06g076570.1.1	AAGATTTAGACTTCCGGAGAATGC	GCCCATACAAGGATACAATAAATTCACAT
SlHSP20.1	AJ225046	Solyc06g076540.1.1	CTGTTCCAAAGGAAGAAGTGAAGA	AGAAACAGCGACAACTCAAACACT
ACS6	NM_001247235.2	Solyc08g008100.2.1	GGGAGTTGAAGTTGCAACAAA	CCTTGCCTGGACCATAGGAGA
NR	NM_001246965	Solyc09g075440.2.1	AGAATATTGCTTTGGATGTAGC	GAGAGCACAGAGCAATAACTGCA
TAG11	NM_001247265.2	Solyc11g028020.1.1	CATGATGCCATCTGGTGGGG	CCTTTTGTGATCAGGAGACAATGGA
TAG1	NM_001308296.2	Solyc02g071730.2.1	GTGCCTCCACCTCAGCAATT	GGGGTTGGTCTTGTCTAGGGT
RIN	NM_001247047.1	Solyc05g056620.1.1	TCGAGAAAGAACCAACTCATGCA	GTTTGCTGGTGCCATTCACC
PG	NM_001247092	Solyc10g080210.1.1	GTGGTCCAGGTCATGGTATAAGTATTGG	GATCCTAACTCCATTTTCGGCACC
SR3L	XM_010316362.1	Solyc12g099340.1.1	TCCGGAGTAATGACAGTGAGCA	GATTCGGGAGTTGGATCGTCTA

Supplementary Table S2.
CArG box motifs found in the promoter of small heat shock protein genes (*sHSPs*)

Gene Name	SGN Database Gene Locus	CArG Motif Positions in (-)3Kb Promoter Region	CArG Motif Sequence	PLACE Motif Nomenclature	Motif Type
SlHSP17.6	Solyc06g076560.1.1	(-)2221	CAAAAAAAAG	$CARGCW_8GAT$	Atypical $[C(A/T)_8G]$
		(-)2306	CTTATTTATG	$CARGCW_8GAT$	Atypical $[C(A/T)_8G]$
		(-)2345	CAATATTTAG	CARGCW8GAT	Atypical $[C(A/T)_8G]$
		(-)2686	CTATAATAAG	$CARGCW_8GAT$	Atypical $[C(A/T)_8G]$
SlHSP20.0	Solyc06g076570.1.1	(-)303	CTAAATTTTG	$CARGCW_8GAT$	Atypical $[C(A/T)_8G]$
		(-)917	CTTAATTATG	$CARGCW_8GAT$	Atypical $[C(A/T)_8G]$
		(-)1047	CATTATTTTG	$CARGCW_8GAT$	Atypical $[C(A/T)_8G]$
		(-)2014	CATATATATG	$CARGCW_8GAT$	Atypical $[C(A/T)_8G]$
SlHSP20.1	Solyc06g076540.1.1	(-)901	CATATAAATG	$CARGCW_8GAT$	Atypical $[C(A/T)_8G]$
		(-)994	CAAAATTTTG	$CARGCW_8GAT$	Atypical $[C(A/T)_8G]$
		(-)1323	CATATTTTTG	$CARGCW_8GAT$	Atypical $[C(A/T)_8G]$
		(-)1341	CCATTTAAGG	CARGAT CONSENSUS	Possible $[C(C/T)(A/T)_6(A/G)G]$
		(-)1933	CCAAAAAAGG	CARGAT CONSENSUS	Possible $[C(C/T)(A/T)_6(A/G)G]$

Supplementary Table S3.
Primers pairs specific to the CArG-box/CArG-box-like sites present in the three *sHSP* promoters used in CHIP enrichment assay

Gene	Position name	Locations (-)3Kb upstream to ATG	Position includes	Forward Primer (5′-3′)	Reverse Primer (5′-3′)
SlHSP17.6	P1	(-)2221	P1	AAGGAGAAAATTAAGGGGGGAA	AAATAGAATATATTGTGGAATGAAT
	P2	(-)2306	P2 & P3	TATTTTAGCATTTAAGAGGACTCA	CCTTAATTTTCTCCTTTTTGTTAT
	P3	(-)2345			
SlHSP20.0	P1	(-)303	P1	TTAATTGTTCGGTCTGTTGTTCTT	CCACGTCACAGATCATACAAGT
	P2	(-)917	P2	CAAGTTATCTTTGAGTTAATTCATTA	AACCCAAACTACTTCTACTGC
	P3	(-)1047	P3	TGCTATTATTATGTTCCCCATG	TTGTTTTAGCGGTACCTAAAATT
	P4	(-)2014	P4	CAGCAAGGGCTAAACATGAAAA	CACACCATCATCCATGAAGAATT
*SlHSP*20.1	P1	(-) 901	P1	GGTTGATTTATGGGTCGGATTA	CTTATGGACGTTAGTTGCCATGTT
	P2	(-) 994	P2	GAATATTTGACATTAATTTTATAC	TAATCCGACCCATAAATCAACC
	P3	(-) 1323	P3 & P4	CAATGAAGTTGAAAGAGGAATTC	CAACAAATCCCAAAAATGAGT
	P4	(-)1341			
	P5	(-)1933	P5	GGCTCTGTTATTACTGGACAAGTG	TGGCTAACAATCTTATGCTTCCT
